# Anemia and Hematopoietic Factor Deficiencies in Patients after Endoscopic Gastrostomy: A Nine-Year and 472-Patient Study

**DOI:** 10.3390/nu12123637

**Published:** 2020-11-26

**Authors:** Mariana Brito, Ana Laranjo, Gonçalo Nunes, Cátia Oliveira, Carla Adriana Santos, Jorge Fonseca

**Affiliations:** 1 GENE–Artificial Feeding Team, Gastroenterology Department, Hospital Garcia de Orta, 2805-267 Almada, Portugal; goncalo.n@hotmail.com (G.N.); sofi.doliveira@gmail.com (C.O.); carla.adriana.santos@hotmail.com (C.A.S.); jorgedafonseca@hotmail.com (J.F.); 2 PaMNEC–Grupo de Patologia Médica, Nutrição e Exercício Clínico, CiiEM, Centro de Investigação Interdisciplinar Egas Moniz, 2829-511 Monte da Caparica, Portugal; 3 Gastroenterology Department, Hospital do Espírito Santo de Évora, 7000-811 Évora, Portugal; anamlaranjo@gmail.com

**Keywords:** anemia, hematopoietic factors, gastrostomy, PEG

## Abstract

Introduction and aims: Patients undergoing percutaneous endoscopic gastrostomy (PEG) may present protein-energy malnutrition, anemia and deficiencies of hematopoietic factors, e.g., iron, folate and vitamin B12. There are no comprehensive studies on anemia or other hematological changes in PEG-patients. Our aim was to evaluate the hematological status of dysphagic patients that had undergone PEG and its association with clinical outcome. Methods: This research comprises a retrospective study of patients followed by our Artificial Feeding Team, submitted to PEG from 2010 to 2018. Patients were divided into two etiological groups: neurological dysphagia (ND) and head/neck or esophageal disorders (HNE). Laboratory data included serum albumin, hemoglobin, mean corpuscular volume, ferritin, transferrin, iron, vitamin B12 and folate. Survival after PEG was recorded in months, until death or December 2018. Results: We evaluated 472 patients; 250 (53%) presented anemia at the moment of gastrostomy, mostly normocytic (*n* = 219), with laboratory data suggestive of anemia of chronic disease (ACD). Six patients (1.3%) presented vitamin B12 deficiency and 57 (12.1%) presented folate deficit. No statistically significant difference in hemoglobin was found between the etiological groups (*p* = 0.230). Folate and vitamin B12 levels were lower in the HNE group (*p* < 0.01). A positive correlation between hemoglobin and survival was present (*p* < 0.01, *r* = 0.289), and hemoglobin levels were lower in the deceased population (*p* < 0.01). Conclusion: Anemia is frequent in PEG-patients, mostly with the features of ACD or multifactorial. It is associated with significant decrease in survival and may be viewed as a marker of severe metabolic distress, signaling poor outcome.

## 1. Introduction

Anemia is defined as low hemoglobin concentration [1,2]. Causes include a lack of nutrients, such as iron, vitamin B12 and folate. Iron is a major substrate for erythropoiesis, and its regulation involves transferrin, the major iron plasma transporter, and ferritin [3,4]. Vitamin B12 and folate are required for blood cell formation, but a balanced diet fulfils daily requirements.

Inflammatory, infectious or malignant disorders can cause anemia, referred to as anemia of chronic disease (ACD) [5,6]. ACD is characterized by reduced iron availability, and is mostly normocytic and normochromic, although it can be microcytic and hypochromic in less than 25 percent of patients [5,7]. Serum iron and transferrin are reduced in ACD. Serum ferritin is normal or elevated in ACD, and represents a poor marker of iron stores in chronic inflammation, since ferritin is a positive acute phase reactant (APR).

When oral intake is insufficient to meet a patient’s needs, tube feeding is recommended. Percutaneous endoscopic gastrostomy (PEG) is the gold standard for long-term enteral feeding if inadequate oral intake exceeding three weeks is expected [8]. In recent years, the number of PEG-feeding patients has been increasing, mostly comprising patients suffering from dysphagia due to head or neck cancer and neurological disorders [9]. When referred for PEG, patients often present long-standing reduced food intake and protein-energy malnutrition (PEM), which may contribute to anemia [10].

To the best of our knowledge, there are no comprehensive studies on anemia or hematological changes in PEG-fed patients. The present study aims to evaluate the hematological status of dysphagic patients that had undergone endoscopic gastrostomy and its association with poor clinical outcome. Our objectives were

1. To evaluate the prevalence and characterize anemia in patients that underwent PEG;
2. To identify deficiencies of hematopoietic factors, namely, iron, folate and vitamin B12;
3. To compare the prevalence of anemia and deficiencies of hematopoietic factors between patients with head/neck cancer or esophageal disorders and those with neurological dysphagia;
4. To study the association between anemia and patient outcome after PEG.

## 2. Methods

A retrospective analysis of the PEG patient database was performed for all adults submitted to endoscopic gastrostomy from January 2010 to December 2018 and followed by our Artificial Feeding Team (GENE) at the Hospital Garcia de Orta. The only initial exclusion criteria was age under 18 years. All subjects and/or their legal caregivers were informed and gave their informed consent.

The following clinical data was collected for each patient: age, gender, clinical indication for PEG, Body Mass Index (BMI), date of gastrostomy and current patient status (deceased, lost to follow-up, alive and still PEG-fed, or alive and having resumed oral feeding). For deceased patients, the date of death was recorded, and survival was calculated in months after the procedure. For the remaining patients, survival was calculated in months from the gastrostomy until December 2018. Serum levels of Albumin, Hemoglobin, Mean Corpuscular Volume (MCV), Ferritin, Transferrin, Iron, Vitamin B12 and Folate were also evaluated. Anthropometric and laboratorial data were obtained using the clinical files of the Artificial Feeding Team. Patients without all the necessary data were excluded.

All patients underwent PEG after a 12 h fast. Antithrombotic therapy was managed according to guidelines [11]. No patient received intravenous intensive fluid therapy in the 72 h before the procedure.

Patients were divided into two etiological groups according to the underlying disease leading to dysphagia: head/neck cancer or esophageal disorders (HNE) and neurological dysphagia (ND).

The body mass index of most patients was obtained using the equation weight/height^2^. If patients were unable to stand up for weight and height evaluation, BMI was estimated using the Mid Upper Arm Circumference (MUAC), and regression equations described by Powell-Tuck and Hennessy [12], which were previously proved to provide a reliable BMI estimation in PEG patients [13,14]. A BMI < 18.5 kg/m^2^ for adult patients younger than 65 years and <22 kg/m^2^ for patients 65 years old or older was considered to be indicative of malnutrition [15].

A blood sample was obtained just before the gastrostomy procedure, as part of the nutritional evaluation. Albumin under 3.5 g/dL and transferrin under 200 mg/dL were considered to be suggestive of inflammation and/or malnutrition. Normal cutoff values were evaluated, according institutional protocol of our laboratory:

Albumin: 3.5–5.0 g/dL
Hemoglobin: 120–180 g/L
MCV: 80–100 fL
Transferrin: 200–360 mg/dL
Iron: 45–160 mcg/dL
Ferritin: 40–200 ng/mL
Vitamin B12: 197–866 pg/mL
Folate: 4.6–34.8 ng/mL

Statistical analysis was performed using the Statistical Package for Social Sciences (IBM SPSS^®^ Statistics, version 22.0, Chicago, IL, USA). Categorical variables were presented as frequencies and percentages and continuous variables as means and standard deviations (SD), or medians and interquartile ranges (IQR) for variables with skewed distributions. Normal distribution was checked using Shapiro-Wilk test or skewness and kurtosis. Nonparametric Spearman’s correlation and Mann-Whitney tests were applied to compare variables without normal distribution, and a Parametric Independent T-test was used to compare normally distributed variables. A Chi-square test was used for categorical variables. All reported *p* values are two-tailed, with a *p*-value below 0.05 indicating statistical significance.

## 3. Results

A total of 601 adults were submitted to PEG between 2010–2018. Among these, 129 (21.5%) were excluded due to incomplete data. Finally, 472 patients were enrolled (294 males (62.3%) and 178 females (37.7%)) aged 22–100 years with a median age of 70 years (IQR = 22).

The clinical indications for PEG were divided into two etiological groups: neurological dysphagia (ND group) (65.3%) and head/neck or esophageal disorders (HNE group) (34.7%), mostly cancer or stenosis after cancer therapy. Regarding clinical outcome, 270 patients (57.2%) died after PEG, with a median survival of five months (IQR 14) and a median age at time of death of 71 (IQR 22); 110 (23.3%) were lost to follow-up; 87 (18.4%) were alive and still PEG fed, and 5 (1.1%) were alive and had resumed oral intake. For the nondeceased patients, the median survival from the date of PEG placement until December 2018 was 29 months (IQR 51). The main characteristics of the population are described in Table 1.

Patients in the HNE group were younger (HNE: median age 61.5 years, IQR 14; ND: median 75, IQR 19, *p* < 0.01). In the HNE group, most patients were males (84.1%), and only 15.9% were female. In the ND group, the number of males and females was similar (50.6% vs. 49.6%). There was an association between gender and the HNE group, with men being more likely to belong to that group (*p* < 0.01). No association was found regarding survival in the two groups (*p* = 0.908).

At the moment of the gastrostomy procedure, 224 patients (47.5%) presented low albumin, and 295 patients (62.5%) presented low transferrin. A total of 190 patients (40.3%) presented both low albumin and transferrin. A total of 207 patients (43.9%) presented low BMI (56 under 65 years, 151 who were 65 years or older). The results of the initial nutritional assessment are described in Table 2.

On the procedure day, most patients (*n* = 250, 53%) presented anemia. Mostly, 219, presented normocytic anemia (46.4%), whereas 17 (3.6%) showed microcytic anemia and 14 (3%) macrocytic anemia (Table 2). Regarding the 219 patients with normocytic anemia, more than half (*n* = 125, 57%) presented low iron with low/normal transferrin levels, suggesting ACD. Of these, 18 (14.4%) presented normal ferritin levels and 103 (82.4%) presented high ferritin concentration, which were typical findings for ACD. Eighteen patients presented a combination of iron and folate deficiency and one showed a combination of iron, folate and vitamin B12 deficiency, indicating probable multifactorial anemia. Only four patients showed low iron with low ferritin levels, with a low to normal range of transferrin, potentially indicating early-stage iron deficiency anemia. Of the 94 normocytic anemia patients with normal iron levels, only nine presented folate deficiency and none presented vitamin B12 deficit. Among these patients, there was no clear cause for the low hemoglobin and we assumed multifactorial anemia.

Regarding the 17 patients with microcytic anemia, 14 showed low iron with low/normal transferrin. Four of these presented high ferritin as well, a finding that may be indicative of ACD in a microcytic stage. Two of these 17 showed low ferritin levels, which was suggestive of iron deficiency anemia.

Regarding the 14 patients with macrocytic anemia, three presented folate deficiency and one presented vitamin B12 deficiency. Seven presented increased Red Cell Distribution Width (RDW) (six without folate or vitamin B12 deficits, and one with folate deficiency), which may correspond to a period of compensatory reticulocytosis. In four patients, no cause for macrocytosis was identified. The distribution of hematopoietic factor deficiencies in the different types of anemia is presented in Table 3.

Two hundred and twenty-two patients (47%) showed normal hemoglobin levels at the gastrostomy procedure. The hematopoietic factor deficiencies in these patients are described in Table 3.

Among the 472 patients, six (1.3%) presented vitamin B12 deficiency and 57 (12.1%) showed folate deficiency (Table 4). Low vitamin B12 levels were present in two of 293 elderly patients (0.7%) and in four of 179 nonelderly (2.2%). No statistically significant difference was detected when correlating age and vitamin B12 levels (*p* = 0.274). Conversely, low folate levels were present in 26 of 293 elderly patients (8.9%) and in 31 of 179 nonelderly patients (17.3%). A significant positive correlation was found between folate levels and age (*r* = 0.222, *p* < 0.01).

Regarding hemoglobin levels, there was no statistically significant difference between the two etiological groups (*p* = 0.230). There is a positive statistically significant correlation (*p* < 0.01, *r* = 0.289) between hemoglobin levels and survival in the global population (*n* = 472), with patients with lower levels presenting shorter survival. When analyzing the deceased population (*n* = 270), a positive statistically significant correlation (*p* <0.01, *r* = 0.262) was also found. We tested for an association between hemoglobin in deceased and nondeceased patients. In the deceased group, hemoglobin levels were significantly lower (median 115.87, SD 18.251) compared to the nondeceased group (median 126.96, SD 21.162), *p* = 0.000.

Regarding the comparison of the two groups, i.e., HNE (our “cancer group”) and ND, our study did not find significant differences in hemoglobin (*p* = 0.230), ferritin (*p* = 0.094), transferrin (*p* = 0.433) or iron levels (*p* = 0.500). There was a statistically significant difference in folate levels (*p* < 0.01) and vitamin B12 levels (*p* = 0.006) between the two groups, with both values being lower in the HNE group.

## 4. Discussion

PEG is the preferred enteral feeding method if nutritional support is required for more than 3–4 weeks [8]; it improves patient nutritional status, quality of life and overall survival. When patients undergo PEG, their nutritional status should be monitored to detect and correct malnutrition. However, many patients are bedridden and present speech difficulties. The usual nutritional assessment tools with questions and answers may therefore be inadequate. Enteral feeding teams often depend on anthropometric and laboratory data to monitor patient evolution [14,16]. Since nutritional assessments of PEG-fed patients are challenging, serum protein may be useful. Serum proteins such as albumin and transferrin are classic markers for PEM, and have been considered major indicators of malnutrition. The concentrations of these proteins decrease by at least 25% in inflammatory states; however, they are negative APR, and are markers of inflammatory activity, so they should be used with other tools. Nowadays, albumin is considered mostly as a prognosis tool and less as a nutritional indicator. Ferritin is a positive APR whose concentration increases in inflammation [17,18]. APR accompanies both acute and chronic inflammatory states and is associated with a wide variety of disorders, including inflammatory diseases and various tumors. Patients suffering from long-standing dysphagia present a high risk of developing malnutrition due to reduced oral intake and the wasting effects of underlying disorders, including the reduction of the levels of hematopoietic factors, namely iron, folate and vitamin B12. Nearly half of the patients in this study presented anthropometric and/or laboratory values suggestive of PEM. In fact, most patients that underwent endoscopic gastrostomy presented reduced food intake in the weeks or months prior to the procedure, signaling malnutrition risk. Having a clear clinical/nutritional picture of PEG-patients and identifying factors that could help physicians predict poor outcome is of the utmost importance.

In our study, patients in the HNE group were significantly younger than those in ND group; this was probably due to a high prevalence of neurologic disorders in older patients. Most patients in the HNE were male, in accordance with more striking alcoholic and smoking habits in this gender, whereas in the ND group, female and male genders were equivalent.

We identified anemia in about half of the patients at the moment of gastrostomy, mostly normocytic anemia. More than half showed laboratory findings suggestive of ACD (low iron, low/normal transferrin and normal/high ferritin). Of the patients with normal hemoglobin levels, about 25% presented low iron, probably in relation with an initial phase preceding anemia. There was no statistically significant difference in hemoglobin levels between the two etiological groups.

Total stores of vitamin B12 are typically in the range of 2 to 5 mg, i.e., sufficient to supply needs for years. Conversely, folate stores are estimated to be approximately 0.5 to 20 mg, and the capacity to supply patient needs are significantly lower [19,20]. Even when vitamin B12 intake ceases, deficiency does not develop for at least one or two years, sometimes longer. If folate intake ceases, deficiency may develop rapidly, i.e., within weeks to months. Some studies have demonstrated an association between vitamin B12 deficiency and old age. Vitamin B12 deficiency occurs frequently (>20%) among elderly people, but it is often unrecognized because the clinical signs are subtle. The main etiologies are food-cobalamin malabsorption syndrome (>60% of all cases), pernicious anemia (15–20% of all cases), insufficient dietary intake and malabsorption [21,22]. In our study, a small percentage of patients showed vitamin B12 deficiency (1.3%), with only half of them presenting anemia. Although there are studies suggesting an association between vitamin B12 levels and old age, we found no statistically significant correlation. Low folate levels were found in a larger number of patients (12.1%), with more than half presenting anemia. The greater percentage of patients with folate deficiency when compared to vitamin B12 was in accordance with larger stores of vitamin B12, in contrast with folate reserves, which are only sufficient for a short period.

The mean hemoglobin levels were significantly lower in the deceased population compared to the nondeceased group. In fact, anemia was the only hematological change associated with statistically significant decrease in survival. PEG patients often present multifactorial causes for anemia and laboratory data suggestive of ACD. These patients usually present malnutrition and different levels of hematopoietic nutrient deficit due to extended periods of insufficient oral intake. Moreover, they frequently suffer from underlying inflammatory or malignant disorders which are associated with ACD. Anemia in PEG patients may be looked at as a surrogate marker of severe metabolic distress, resulting in poor outcome. However, the presence of concomitant metabolic alterations make it difficult to make accurate estimations of the risk attributable to anemia alone.

This study presented some limitations. It was carried out in a single hospital and the data were dependent on patients’ clinical files, which were not always complete. Patient comorbidities were not considered. The study focused on initial PEG patient assessments, and did not include possible nutritional supplementation during PEG feeding. Intervention in the nutritional status of PEG-fed patients may be an interesting avenue of research in future studies. We are presently starting a prospective study evaluating the hematologic evolution of PEG patients over the first year of PEG feeding; it will include a very large number of patients and seeks to clarify the relationship between anemia and poor outcome of PEG patients.

## 5. Conclusions

Most dysphagic patients undergoing endoscopic gastrostomy present anemia, probably due to a combination of factors such as a lack of nutrients and anemia associated with malignancy/inflammatory states. Also, they frequently present low serum levels of one or more hematopoietic factors, namely iron, folate and vitamin B12. Teams taking care of PEG patients should be aware and prevent or treat deficiencies of hematopoietic factors. Anemia was the only hematological change associated with statistically significant decrease in survival and may be considered a surrogate marker of severe metabolic distress in patients undergoing PEG, resulting in poor outcome.

## Figures and Tables

**Table 1:** Characteristics of the study population (*n* = 472).

Characteristics	*n* = 472
Age (years)	
Median (IQR)	70 (22)
Min	22
Max	100
Gender-*n* (%)	
Female	178 (37.7%)
Male	294 (62.3%)
Etiological groups-*n* (%)	
HNC/Esophageal disorder (HNE)	164 (34.7%)
Female	26 (15.9%)
Male	138 (84.1%)
Age (Median, IQR)	61.5 (14)
Neurological dysphagia (ND)	308 (65.3%)
Female	152 (49.4%)
Male	156 (50.6%)
Age (Median, IQR)	75 (19)
Clinical outcome (*n*, %)	
Deceased	270 (57.2%)
Lost to follow-up	110 (23.3%)
Alive/PEG-fed	87 (18.4%)
Alive/oral-fed	5 (1.1%)

IQR, interquartile range; HNC, head or neck cancer; HNE, head/neck or esophageal disorders; ND, neurological dysphagia; PEG, percutaneous endoscopic gastrostomy.

**Table 2:** Initial nutritional assessment.

Initial Nutritional Assessment	*n* (%)
Albumin and transferrin	
Low albumin	224 (47.5%)
Low transferrin	295 (62.5%)
Low albumin and transferrin	190 (40.3%)
BMI	
Low BMI	207 (43.9%)
<65 years	56
≥65 years	151
Hemoglobin levels	
Anemia	250 (53%)
Normocytic anemia	219
Microcytic anemia	17
Macrocytic anemia	14
Normal hemoglobin	222 (47%)

**Table 3:** Distribution of hematopoietic factor deficiencies.

Hematopoietic Factor Deficiencies	*n*
Normocytic anemia	219
Low iron + low/normal transferrin	125
High ferritin	103
Normal ferritin	18
Low ferritin	4
Iron + folate deficiency	18
Iron + folate + Vitamin B12 deficiency	1
Normal iron	85
Normal iron + folate deficiency	9
Microcytic anemia	17
Low iron+low/normal transferrin	14
High ferritin	4
Low ferritin	2
Macrocytic anemia	14
Folate deficiency	3
Vitamin B12 deficiency	1
Normal hemoglobin levels	222
Normal MCV	206
Low iron	53
Low folate	22
Low vitamin B12	2
High MCV	15
Low folate	2
Low folate + vitamin B12	1

MCV, Mean Corpuscular Volume.

**Table 4:** Prevalence of folate and vitamin B12 deficiency in the study population.

Folate and Vitamin B12 Deficiencies	*n* (%)
Vitamin B12 deficiency	6 (1.3%)
Median age 60.5 years, IQR 16	
Normal hemoglobin levels	3
Normocytic anemia	2
Macrocytic anemia	1
Folate deficiency	57 (12.1%)
Median age 60 years, IQR 23	
Normocytic anemia	27
Normal hemoglobin levels	25
Macrocytic anemia	4
Microcytic anemia	1

IQR, Interquartile range.
